# Sex differences in effect of patients-centered intervention on blood pressure in patients with hypertension

**DOI:** 10.1038/s41598-023-41286-z

**Published:** 2023-08-25

**Authors:** Hyun-Sun Kim, Hyun-Jin Kim

**Affiliations:** 1 Department of Nursing, College of Nursing, Eulji University, Uijeongbu-si, Gyeonggi-do Republic of Korea; 2 Division of Cardiology, Department of Internal Medicine, Hanyang University College of Medicine, Hanyang University Guri Hospital, 153, Gyeongchun-ro, Guri-si, Gyeonggi-do 11923 Republic of Korea

**Keywords:** Health services, Patient education, Health care, Cardiology, Cardiovascular biology

## Abstract

Hypertension exhibits sex-related differences in its causes, symptoms, and complications. In this study, we aimed to confirm the efficacy of hypertension intervention by applying a patient-centered approach based on sex differences. We enrolled 95 hypertensive patients in this prospective quasi-experimental pretest–posttest study. The patient-centered lifestyle intervention included penalized nutrition and exercise education in 30-min one-on-one sessions. Before the intervention, we conducted a pretest to evaluate physical examination, behavioral status, quality of life (QoL), blood pressure (BP) measurements, and routine blood tests. The same evaluations were conducted again in a posttest after 3 months. After 3 months of patient-centered intervention, all patients showed a decrease in systolic BP by 2.87 mmHg and diastolic BP by 1.04 mmHg. However, there was no significant difference in BP between men and women after the 3-month follow-up. There were differences in lipid profiles based on sex, with total cholesterol and low-density lipoprotein cholesterol levels decreasing in men and increasing in women. Behavioral and QoL scores improved after the intervention; however, there was no significant difference based on sex. A patient-centered lifestyle intervention for hypertensive patients can effectively lower BP, and sex-specific risk factors affecting its efficacy have been identified.

## Introduction

Hypertension is a common chronic disease and a significant risk factor that increases the risk of severe complications, such as cardiovascular disease and stroke^1^. According to the fact sheet released by the Korean Society of Hypertension in 2021, hypertension is gradually increasing among adults aged 30 years and older; 31.1% of men and 22.8% of women were found to have hypertension^2^. It has been revealed that sex-specific etiologies, signs, and risk factors for cardiovascular complications are associated with hypertension; specifically, awareness, treatment, and adherence to hypertension management differ between men and women^3^. According to the fact sheet released by the Korean Society of Hypertension, the proportion of patients with controlled hypertension was < 48%. The hypertension control rate for women was higher than that for men (49% vs. 47%) before the age of 60. After the age of 60, the hypertension control rate for men was higher than that for women (66% vs. 51%), indicating a clear sex difference^2^. However, despite the sex differences in the prevalence and control rate of hypertension, previous studies have shown conflicting results regarding risk factors, cardiovascular complications, treatment, and interventions for hypertension based on sex; thus, the differences in hypertension management by sex have not been established^3–5^. Furthermore, only a few studies have investigated the factors that influence the treatment and control of hypertension following nursing interventions based on sex.

The importance of lifestyle modifications in treating hypertension has been continuously emphasized in domestic and international guidelines, in addition to the pharmacological treatment of hypertensive patients. Previous studies have reported positive changes in physical, behavioral (health behaviors), and psychological (quality of life (QoL), self-efficacy) aspects, and the effects on blood pressure (BP) control, through lifestyle modification using nursing interventions^6–8^. Lifestyle modification through nursing interventions integrates patient-centered care by considering individual preferences, needs, and values^9^. It emphasizes collaboration between patients, caregivers, and healthcare providers, and encourages patient engagement in treatment planning and lifestyle changes^9, 10^. Specifically, interventions focused on healthy eating habits and exercise can be considered the most cost-effective methods for patients with hypertension^7, 11^. Considering that patients with hypertension were less likely to practice healthy lifestyles^12^, the need for interventions that specifically focus on diet and exercise is strongly emphasized. Providing patient-centered interventions that reflect the individual patient’s lifestyle and characteristics using nutrition and exercise can enhance the relationship between patients and nurses, share information and knowledge, and increase the performance of physical intervention activities^13, 14^. In patient-centered nursing intervention studies targeting hypertensive patients, there have been results where the effectiveness of BP reduction was reported by applying patient-centered intervention programs^15–17^. Such programs are customized, considering individual physical, emotional, and social factors, and help promote patient participation and cooperation, leading to active intervention.

Studies that focus on sex differences in hypertension are limited, and in particular, no study has applied patient-centered interventions to hypertensive patients to confirm the sex-specific effects. In this study, we aimed to apply a patient-centered lifestyle improvement intervention (PLI) that includes education on nutrition and exercise to manage BP in hypertensive patients and evaluate its effects on physical and behavioral changes and quality of life quality of life. We equally aimed to analyze whether there were any differences based on sex.

## Results

### Baseline characteristics

Table 1 shows the baseline characteristics of the patients according to sex. Women were significantly older than men and had a lower body mass index (BMI). Number of men who smoked and consumed alcohol was significantly higher than women. However, the two groups had no significant differences in previous medical history or medication status, including antihypertensive and lipid-lowering drugs. All patients were receiving antihypertensive drugs, and none had changed their medications for 3 months. Furthermore, the two groups had no significant difference in baseline systolic blood pressure (SBP), diastolic blood pressure (DBP), or heart rate (HR).

**Table 1:** Baseline characteristics.

	All (n = 95)	Men (n = 55)	Women (n = 40)	*p*-value
Age, years	63.39 ± 13.22	60.11 ± 13.20	67.90 ± 11.99	0.004
Height, cm	161.89 ± 8.93	167.13 ± 6.98	154.68 ± 5.74	< 0.001
Weight, Kg	67.50 ± 13.95	73.62 ± 13.13	59.09 ± 10.25	< 0.001
BMI, Kg/m^2^	25.58 ± 3.76	26.23 ± 3.43	24.69 ± 4.05	0.049
SBP, mmHg	130.03 ± 17.23	131.96 ± 18.03	127.38 ± 15.91	0.202
DBP, mmHg	74.69 ± 12.43	76.36 ± 12.89	72.40 ± 11.53	0.126
HR, bpm	75.81 ± 16.63	77.18 ± 16.54	73.93 ± 16.78	0.349
Previous medical history, n (%)				
Diabetes	32 (28.1)	20 (36.4)	12 (30.0)	0.522
Dyslipidemia	43 (37.7)	24 (43.6)	19 (47.5)	0.712
CAD	47 (41.2)	29 (52.7)	18 (45.0)	0.462
HF	13 (11.4)	7 (12.7)	6 (15.0)	0.753
Smoking, current	23 (20.2)	22 (40.0)	1 (2.5)	< 0.001
Alcohol drinking	40 (48.2)	37 (67.3)	3 (7.5)	< 0.001
Live with family	73 (64.0)	41 (74.5)	32 (80.0)	0.539
Live alone	22 (19.3)	14 (25.5)	8 (20.0)	
Medication, n (%)				
Antihypertensive drug				–
ARB or ACEi	52 (54.7)	29 (52.7)	23 (57.5)	0.681
CCB	50 (52.6)	28 (50.9)	22 (55.0)	0.835
BB	49 (51.6)	28 (50.9)	21 (52.5)	1.000
Thiazide	13 (13.7)	9 (16.4)	4 (10.0)	0.282
Lipid-lowering drug	61 (64.2)	36 (65.5)	25 (62.5)	0.830

*ACEi* angiotensin-converting enzyme inhibitor, *ARB* angiotensin receptor blocker, *BB* beta-blocker, *BMI* body mass index, *CAD* coronary artery disease, *CCB* calcium channel blocker, *DBP* diastolic blood pressure, *HF* heart failure, *HR* heart rate, *SBP* systolic blood pressure.

### Changes in blood pressure and lipid profile after patients centered intervention

**Figure 1:**
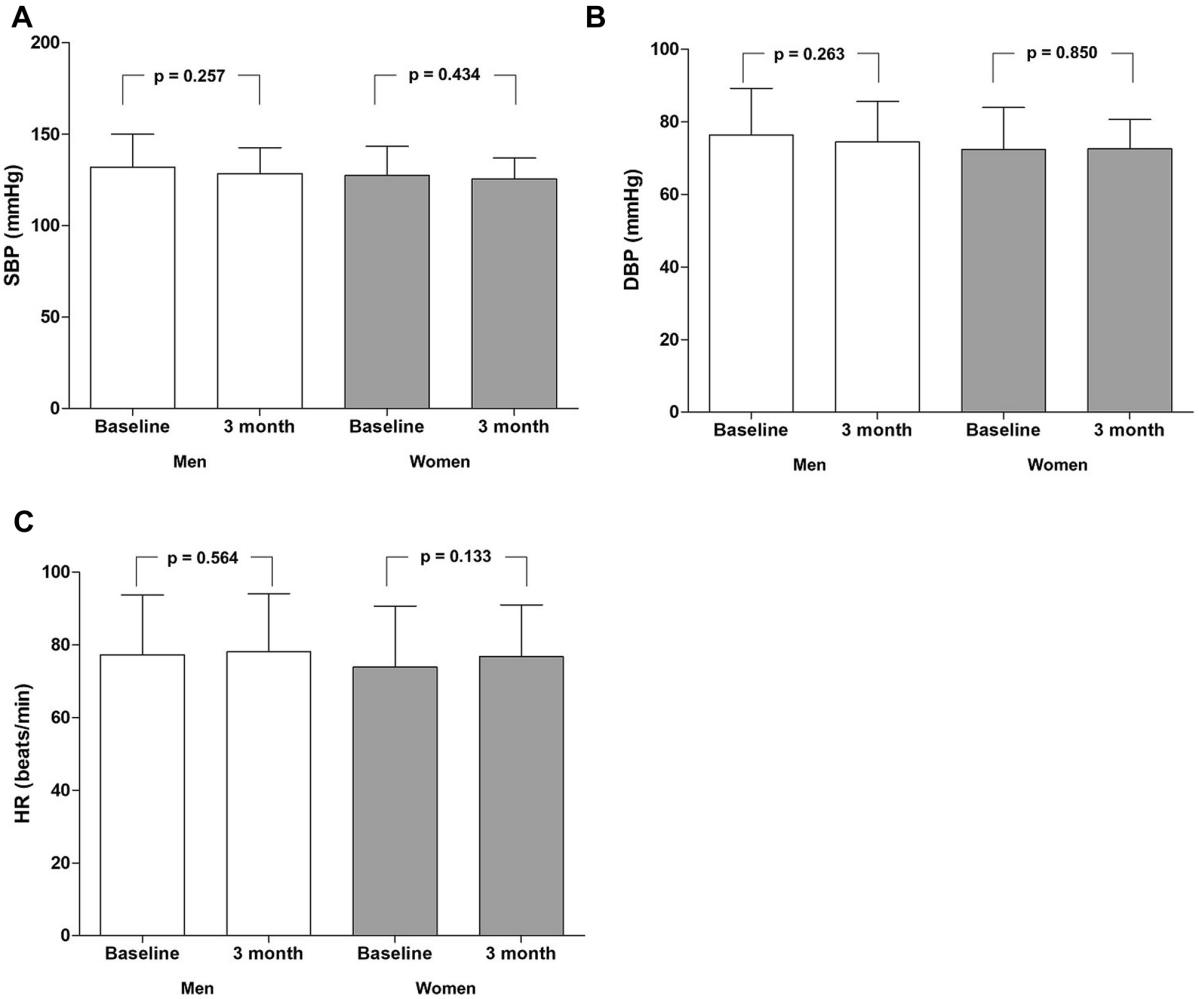
Changes in blood pressure and heart rate after the patient-centered intervention. (**A**) Changes in SBP after 3 months. Changes in SBP after 3 months in men were not significant compared to baseline, and also in women, (**B**) Changes in DBP after 3 months. Changes in DBP after 3 months in men were insignificant compared to baseline, and also in women, (**C**) Changes in HR after 3 months. Changes in HR after 3 months in men were insignificant compared to baseline and women. *DBP* diastolic blood pressure, *HR* heart rate, *SBP* systolic blood pressure.

Figure 1 shows the changes in SBP, DBP, and HR 3 months after the patient-centered intervention. SBP decreased by 2.87 mmHg, and DBP decreased by 1.04 mmHg in all patients after the patient-centered intervention. Changes in BP (Fig. 1A,B) and HR (Fig. 1C) at baseline and after 3 months did not significantly differ in both men and women. No significant differences were observed in BP and HR between men and women after 3 months of follow-up (Table 2). Additionally, the degree of changes in SBP, DBP, and HR after 3 months and at baseline did not significantly differ between men and women (SB*p* − 3.64 ± 20.31 mmHg vs. − 1.83 ± 14.83 mmHg, *p* = 0.633; DBP − 1.95 ± 13.97 mmHg vs. 0.20 ± 10.86 mmHg, *p* = 0.420).

**Table 2:** Sex differences in changes in blood pressure after patient-centered intervention.

	All (n = 95)	Men (n = 55)	Women (n = 40)	*p*-value
SBP baseline, mmHg	130.03 ± 17.23	131.96 ± 18.03	127.38 ± 15.91	0.202
SBP, 3-month, mmHg	127.16 ± 13.08	128.33 ± 14.20	125.55 ± 11.34	0.310
Difference in SBP, mmHg	− 2.87 ± 18.14	− 3.64 ± 20.31	− 1.83 ± 14.83	0.633
DBP baseline, mmHg	74.69 ± 12.43	76.36 ± 12.89	72.40 ± 11.53	0.126
DBP, 3-month, mmHg	73.65 ± 9.98	74.42 ± 11.18	72.60 ± 8.07	0.360
Difference in DBP, mmHg	− 1.04 ± 12.73	− 1.95 ± 13.97	0.20 ± 10.86	0.420
HR baseline, mmHg	75.81 ± 16.63	77.18 ± 16.54	73.93 ± 16.78	0.349
HR, 3-month, mmHg	77.54 ± 15.21	78.07 ± 16.02	76.80 ± 14.19	0.689
Difference in HR, mmHg	1.73 ± 12.91	0.89 ± 13.91	2.88 ± 11.46	0.462

*DBP* diastolic blood pressure, *HR* heart rate, *SBP* systolic blood pressure.

The difference in BMI between men and women was significant at baseline and 3 months; however, no significant difference was observed in the degree of change in BMI (Table 3, Supplementary Fig. S1A). Changes in total cholesterol levels were not significantly different at baseline and 3 months in both men and women (Supplementary Fig. S1B). However, the degree of change in total cholesterol showed a significant difference between men and women (− 11.27 ± 48.41 mg/dL vs. 8.40 ± 35.30 mg/dL, *p* = 0.018) (Table 3). After 3 months, the total cholesterol level decreased in men, whereas it increased in women. Additionally, the LDL-cholesterol level was decreased in men and increased in women after 3 months (Supplementary Fig. S1C), which showed a significant difference between men and women (− 11.33 ± 36.98 mg/dL vs. 4.65 ± 22.83 mg/dL, *p* = 0.007) (Table 3). However, there were no significant differences in the degree of change in triglyceride and HDL cholesterol levels between men and women after 3 months (Supplementary Fig. S1D,E).

**Table 3:** Sex differences in changes in lipid profile after patient-centered intervention.

	All (n = 95)	Men (n = 55)	Women (n = 40)	*p*-value
BMI, baseline, Kg/m^2^	25.58 ± 3.76	26.22 ± 3.43	24.69 ± 4.05	0.049
BMI, 3-month, Kg/m^2^	25.51 ± 3.77	26.26 ± 3.33	24.48 ± 4.12	0.022
Difference in BMI, Kg/m^2^	− 0.07 ± 1.01	0.03 ± 1.06	− 0.21 ± 0.95	0.673
T.chol, baseline, mg/dL	156.21 ± 47.01	158.35 ± 48.98	153.28 ± 44.59	0.606
T.chol, 3-month, mg/dL	153.22 ± 38.17	147.07 ± 33.80	161.68 ± 42.47	0.065
Difference in T.chol, mg/dL	− 2.99 ± 44.26	− 11.27 ± 48.41	8.40 ± 35.30	0.018
TG, baseline, mg/dL	151.72 ± 93.42	169.71 ± 107.86	126.98 ± 62.00	0.017
TG, 3-month, mg/dL	131.72 ± 69.28	144.42 ± 80.01	114.25 ± 46.53	0.023
Difference in TG, mg/dl	− 20.0 ± 80.97	− 25.29 ± 99.49	− 12.73 ± 44.74	0.848
LDL-C, baseline, mg/dl	90.58 ± 33.93	93.01 ± 35.99	87.25 ± 31.01	0.417
LDL-C, 3-month, mg/dl	85.98 ± 28.18	81.67 ± 24.90	91.90 ± 31.53	0.081
Difference in LDL-C, mg/dL	− 4.60 ± 32.63	− 11.33 ± 36.98	4.65 ± 22.83	0.007
HDL-C, baseline, mg/dL	50.43 ± 13.75	47.53 ± 12.18	54.43 ± 14.90	0.015
HDL-C, 3-month, mg/dL	53.03 ± 14.70	51.00 ± 14.63	55.83 ± 14.53	0.115
Difference in HDL-C, mg/dL	2.60 ± 10.12	3.47 ± 11.30	1.40 ± 8.23	0.327

*BMI* body mass index, *HDL-C* high-density lipoprotein cholesterol, *LDL-C* low-density lipoprotein cholesterol, *T. Chol* total cholesterol, *TG* triglyceride.

### Blood pressure response to patients centered intervention and the predictors of non-response

Among the 95 patients, 37 (38.9%) responded to patient-centered interventions (Table 4). A change in BP < 10 mmHg was observed in the remaining 58 patients (61.1%). No significant difference was observed in response rate between men and women (41.8% vs. 35.0%, *p* = 0.501).

**Table 4:** Response rate after patient-centered intervention.

	All (n = 95)	Men (n = 55)	Women (n = 40)	*p*-value
Non-Responder^†^	58 (61.1)	32 (58.2)	26 (65.0)	0.501
Responder*	37 (38.9)	23 (41.8)	14 (35.0)	–

^†^Differences of less than 10 mmHg for systolic blood pressure or less than 10 mmHg for diastolic blood pressure.

*Differences of 10 mmHg or more than 10 mmHg for systolic blood pressure or differences of 10 mmHg or more than 10 mmHg for diastolic blood pressure.

According to the univariate analysis by sex (Table 5), old age, lower hemoglobin level, and increased creatinine level of 1 mg/dL or more were associated with BP non-response to patient-centered intervention in men; additionally, dyslipidemia showed a tendency to be associated with BP non-response. Dyslipidemia was independently associated with a 7.19–fold increased hazard for BP non-response to patients centered intervention (hazard ratio [HR] 7.191, 95% confidence interval [CI] 1.543–33.516, *p* = 0.012) after adjustment for all of the possible confounding factors using regression analysis. In men, an increased creatinine level of 1 mg/dL or more showed an independent association with a 5.21-fold increased hazard (HR 5.214, 95% CI 1.077–25.236, *p* = 0.040). In women, living alone and receiving calcium channel blockers (CCB) tended to be associated with BP non-response in univariate analysis. After adjustment for all possible confounding factors, living alone was independently associated with an 8.61-fold increased hazard for BP non-response to patients centered intervention (HR 8.606, 95% CI 1.162–63.749, *p* = 0.035).

**Table 5:** Predictors of non-response to patient-centered intervention.

	Univariate			Multivariate		
	HR	95% CI	*p*-value	HR	95% CI	*p*-value
Men						
Age, years	1.050	1.003–1.100	0.035	1.027	0.963–1.095	0.421
BMI > 25 kg/m^2^	1.282	0.430–3.820	0.656			
Diabetes	1.125	0.368–3.439	0.836			
Dyslipidemia	2.590	0.839–7.998	0.098	7.191	1.543–33.516	0.012
CAD	1.039	0.355–3.038	0.944			
HF	0.952	0.192–4.732	0.952			
Live alone	0.640	0.189–2.170	0.474			
Smoking, current	0.571	0.191–1.710	0.317			
Alcohol	0.835	0.265–2.636	0.759			
ARB or ACEi	0.567	0.191–1.683	0.307			
CCB	1.236	0.423–3.615	0.698			
BB	0.917	0.314–2.678	0874			
Thiazide	0.293	0.065–1.326	0.111			
Lipid-lowering drug	1.414	0.460–4.345	0.545			
Hb, g/dL	0.559	0.324–0.963	0.036	0.613	0.320–1.173	0.139
Creatinine > 1 mg/dL	3.810	1.218–11.919	0.022	5.214	1.077–25.236	0.040
eGFR, mL/min per 1.73 m^2^	0.974	0.949–1.000	0.053			
Na, mmol/L	0.988	0.945–1.033	0.584			
Women						
Age, years	1.018	0.963–1.075	0.528	1.075	0.995–1.161	0.067
BMI > 25 kg/m^2^	0.833	0.222–3.122	0.787			
Diabetes	1.111	0.267–4.632	0.885			
Dyslipidemia	0.857	0.233–3.148	0.816			
CAD	1.800	0.473–6.850	0.389			
HF	1.091	0.174–6.851	0.926			
Live alone	4.259	0.838–21.644	0.081	8.606	1.162–63.749	0.035
Alcohol	1.083	0.090–13.113	0.950			
ARB or ACEi	0.648	0.170–2.470	0.525			
CCB	3.400	0.873–13.239	0.078	4.880	0.952–25.015	0.057
BB	1.167	0.318–4.284	0.816			
Lipid-lowering drug	2.250	0.590–8.579	0.235			
Hb, g/dL	1.033	0.629–1.696	0.899			
Creatinine > 1 mg/dL	2.364	0.238–23.483	0.463			
eGFR, mL/min per 1.73 m^2^	0.993	0.962–1.026	0.686			
Na, mmol/L	1.199	0.886–1.623	0.240			

*ACEi* angiotensin-converting enzyme inhibitor, *ARB* angiotensin receptor blocker, *BB* beta blocker, *BMI* body mass index, *CAD* coronary artery disease, *HF* heart failure, *Hb* hemoglobin, *eGFR* estimated glomerular filtration rate.

### Sex differences in behavioral and quality of life changes

No significant differences were observed in the baseline total behavior score (*p* = 0.147), diet behavior (*p* = 0.173), or exercise behavior (*p* = 0.699) between men and women in terms of their behavioral status (Supplementary Table S2). However, the baseline score for medication behavior showed a significant difference between men and women (*p* = 0.005).

After 3 months, the behavioral status scores increased after intervention by 24.98 ± 4.16 for men and 19.38 ± 3.73 for women. In each area, men scored 7.87 ± 1.99 in diet behavior, and women scored significantly higher at 8.78 ± 1.31 (*p* = 0.014). However, there were no significant differences between men and women in the 3-month exercise and medication behaviors scores. In addition, although behavioral scores increased after the intervention, there were no significant differences in behavioral status scores between men and women after 3 months (*p* = 0.923). When examined in each area, there were no significant differences in the 3-month score changes for diet behavior (*p* = 0.834), exercise behavior (*p* = 0.470), and medication behavior (*p* = 0.069) between men and women.

The baseline QoL scores did not differ significantly between men and women. The QoL scores equally increased after the intervention; however, there were no significant differences between the sexes after 3 months. No significant differences were observed in the QoL scores between men and women (*p* = 0.666) after the intervention.

## Discussion

The patient-centered lifestyle intervention reduced BP by 38.9% after 3 months, with no sex difference among hypertensive patients. In men, dyslipidemia and worsened kidney function were independently associated with BP non-response after the intervention, whereas living alone was independently associated with BP non-response in women. Interestingly, lipid profiles had different findings from BP, as total cholesterol and LDL-cholesterol levels decreased in men and increased in women after 3 months of patient-centered intervention.

We investigated physical effects, behavioral changes, and QoL resulting from PLI, which included nutrition and exercise education, applied to hypertensive patients and identified sex differences. Patient-centered interventions are an essential issue in patients with hypertension, and it is well-known that changing lifestyle habits can decrease BP^10, 18^. In a study by Lee et al.^16^, the efficacy of patient-centered interventions on patients with hypertension was evaluated in terms of BP, medication adherence, health knowledge, dietary habits, self-efficacy, and physical activity. The study showed a reduction in SBP from 132 to 125 mmHg and a reduction in DBP from 78 to 74 mmHg, indicating a significant effect on knowledge, self-efficacy, and lifestyle improvement. Additionally, another study applying patient-centered medication interventions demonstrated a reduction of 3.4 mmHg in SBP^15^. Similar to previous studies, our study’s patient-centered lifestyle improvement intervention reduced SBP by 3.14 mmHg. Although there was no significant difference in the BP reduction by sex, there was a difference in the decrease, with men showing an SBP reduction of approximately 3.63 mmHg and women showing an SBP reduction of approximately 1.83 mmHg.

After applying the PLI, scores for health behavior and QoL improved; however, there was no statistically significant difference between men and women. These results are consistent with previous studies showing that lifestyle modifications, such as dietary and exercise habits, are insufficient for controlling BP in hypertensive patients and that more proactive patient-centered interventions tailored to the individual’s lifestyle are necessary^19, 20^. Additionally, the previous study reveals that weekly text messaging interventions focusing on lifestyle management can significantly control blood pressure in hypertensive patients, a finding that resonates with our research on patient-centered hypertension management^21^. Moreover, our study evaluated the BP-lowering effect of patient-centered lifestyle improvement intervention in hypertensive patients and their behavior and QoL, which is rare in previous studies, and no studies have investigated the differences in intervention effects between men and women.

Lifestyle modifications, such as healthy nutritional habits and exercise, are important for patients with hypertension in addition to medication. The American Heart Association and the European Society of Cardiology guidelines recommend actively promoting such changes to reduce BP^22, 23^. However, sex differences have been observed in hypertension control rates, with different effects even with medication compliance and lifestyle interventions. Young married men have been found to have higher compliance with hypertension management and treatment than young married women^5^. However, older women living alone have more difficulty managing interventions and maintaining BP control^24^. The results of this study suggest that older women living alone may have a higher likelihood of poor BP control and difficulty with intervention management, which is consistent with the findings of our study, indicating an independent association between living alone and the risk of non-responders to patient interventions. Similarly, living with family members can lead to better care and BP management, and it is believed that married men receive more support from their wives than married women regarding health management. Previous studies highlight the importance of patient-centered interventions in hypertension management, focusing on patient empowerment, self-management, and a team approach that includes diet, exercise, medication compliance, and collaboration^25^. These principles resonate with our findings that simple lifestyle changes are not enough, and personalized, proactive interventions, considering sex and individual lifestyles, are needed.

The limitations of this study are as follows: First, as this was a single-center study, careful interpretation is required to generalize the results to all patients. Second, because this study is a quasi-experimental, not a randomized controlled trial for intervention, there are limitations to the effectiveness of the intervention. Furthermore, education in patient-centered lifestyle improvement intervention was only conducted once. Therefore, to verify the beneficial effect, it will be necessary to continue the intervention through future studies or increase the number of participants. Addressing these limitations requires a comprehensive approach that includes digital support, group interventions, app development with features for dietary and exercise tracking, and virtual communities or forums for patients to share experiences. Such an integrated strategy would provide a more engaging and cost-effective way to support hypertensive patients in lifestyle modification, offering continual support and paving the way for innovative, patient-centered care. Finally in our study, we included both longstanding and newly diagnosed hypertension patients, leading to varied attitudes towards lifestyle changes. Longstanding patients may resist modifications due to entrenched beliefs, while the newly diagnosed might be more motivated but face challenges understanding their condition. This difference should be considered when interpreting our results.

Despite the limitations above, the patient-centered lifestyle improvement intervention is significant because it provides nursing intervention tailored to patients' lifestyles, habits, and cultures, respecting them from the patient's perspective rather than the medical staff's perspective. This intervention allowed patients to actively participate in planning and implementing the necessary lifestyle management, such as meals and exercise, and emphasized the importance of family involvement in comparing male and female patients. Compared with traditional one-way education or interventions, this study's approach was effective in helping patients return to their daily lives and recover. This study demonstrated the effectiveness of patient-centered lifestyle improvement interventions in managing BP in hypertensive patients. It is expected to expand the concept of patient-centered interventions to various nursing fields and be widely applied in nursing practice.

In conclusion, patient-centered lifestyle improvement intervention in hypertensive patients can reduce BP by 2.87/1.04 mmHg after 3 months. No difference was observed in the change in BP between men and women according to the intervention. However, there were sex differences in the risk factors that affect the effectiveness of PLI for hypertension. In the future, intervention studies focusing on patients and their families rather than general BP management should be expanded, and interventions tailored to each sex’s specific risk factors should be developed. It requires recognizing sex-specific responses to interventions, as seen with differing risk factors like living alone in women and dyslipidemia in men. This understanding demands the development of tailored strategies that embrace family support and comprehend individual patient requirements. It sets the stage for a sophisticated, patient-centered approach that considers the distinct lifestyles, familial roles, and gender differences, thereby enhancing the effectiveness of interventions for hypertensive patients.

## Methods

### Study design and setting

**Figure 2:**
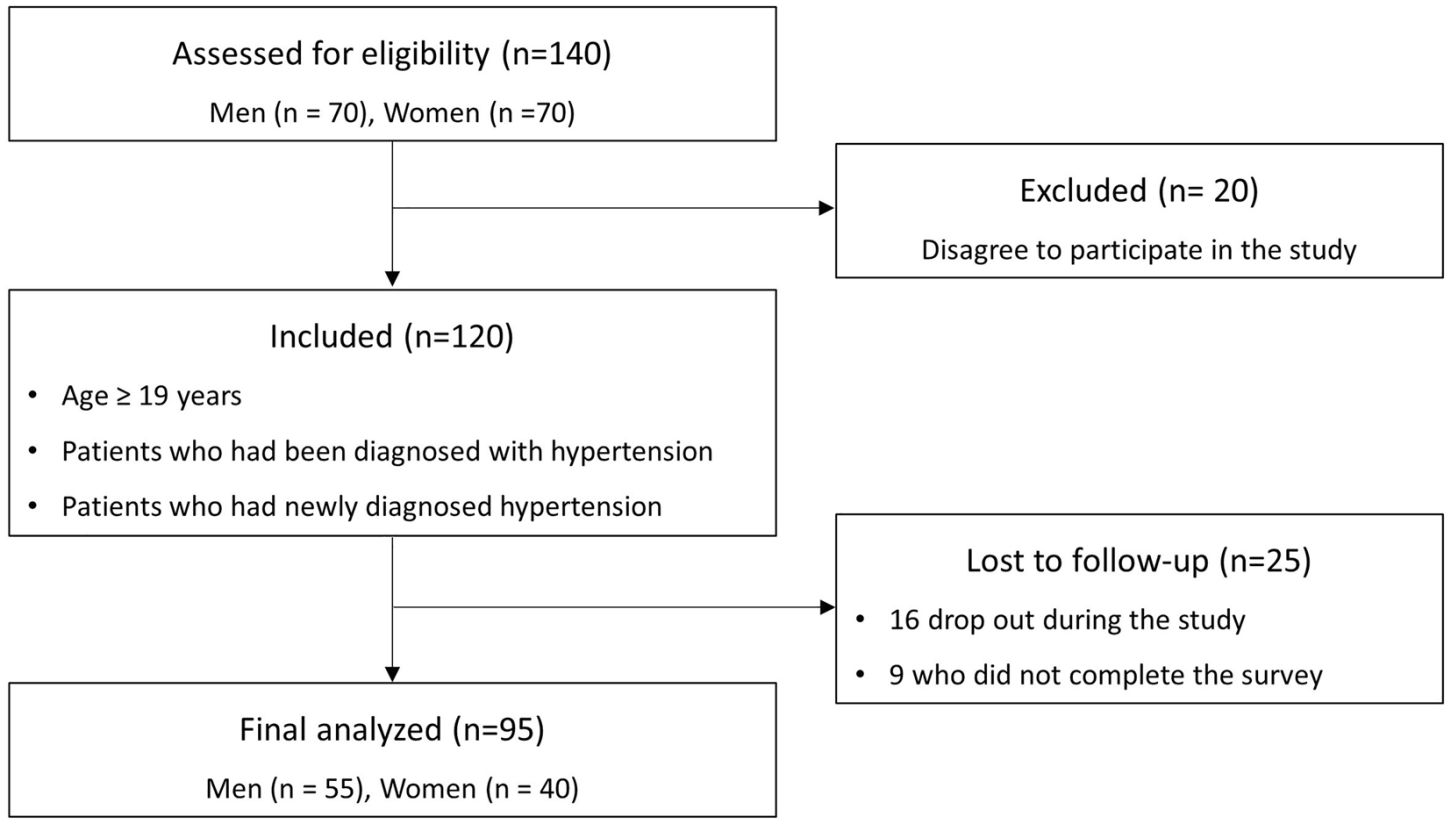
The flow chart of the participants in the study.

This prospective quasi-experimental pretest–posttest study was conducted between July and December 2021 on patients who visited the cardiology department of a general hospital in Gyeonggi Province. Recruitment was conducted through voluntary expression of interest in response to a notice posted on the bulletin board of the cardiology department, inviting those who met the following selection criteria to participate: (1) patients aged 19 years or older. (2) patients who had been diagnosed with hypertension and were receiving antihypertensive medication or patients who had newly diagnosed hypertension and were receiving antihypertensive medication. Patients who met any of the following criteria were excluded from the study: (1) those diagnosed with malignant tumors, (2) those receiving dialysis due to end-stage renal failure. According to a previous study, BP significantly decreased by 3–5 mmHg after lifestyle improvement intervention in patients with hypertension^7^.In this study, 126 patients (63 women and 63 men) were required to test whether there was a difference of 5 mmHg in SBP between men and women after 3 months of patient-centered education with a power of 80% and a significance level of 5%. Considering a dropout rate of 10%, the total number of participants was 140 (70 women and 70 men). After excluding 20 patients who refused to participate, 16 who dropped out during the study, and nine who did not complete the survey, these data of participants were excluded from the final analysis to maintain data integrity. Therefore, 95 patients, including 55 men and 40 women, were analyzed finally (Fig. 2).

The study protocol complied with the Declaration of Helsinki and was reviewed and approved by the Institutional Review Board of Hanyang University Guri Hospital (No. GURI 2021-06-027). All patients provided written informed consent before participating in the study.

## Research hypothesis

The hypothesis of this study asserts that PLI will bring about significant improvements in blood pressure control among hypertensive patients. Specifically, the study aims to identify the differences in systolic and diastolic responses, physical and behavioral changes, and quality of life between male and female participants over a 3-month period of patient-centered education.

### Clinical and laboratory assessments

We conducted a medical history and physical examination, including BP measurements and routine blood tests, on patients with hypertension after the provided consent. Age, sex, height, weight, BMI (kg/m^2^), medical history, including diabetes, hyperlipidemia, coronary artery disease, and heart failure, and social history, including smoking status, alcohol consumption, and medication use were among the clinical features we obtained. Using the UA-767 device from A&D Co., Ltd., Tokyo, Japan, which meets the standards set by the European Hypertension Society, we gauged office BP across all facilities. After a 5-min relaxation period for the patient, we took two BP readings a minute apart and determined the office BP as the average of these two measurements^26^. Blood tests included white blood cell count, hemoglobin, sodium, potassium, blood urea nitrogen (BUN), creatinine, aspartate aminotransferase, alanine aminotransferase, glucose, C-reactive protein, total cholesterol, triglycerides, low-density lipoprotein cholesterol, and high-density lipoprotein cholesterol.

We evaluated changes in participants’ behavioral status and QoL using a self-report format in the pretest and posttest. The behavioral status questionnaire consisted of three parts, nutrition management (two items), exercise management (two items), and medication adherence (two items), comprising six questions. Each item is rated on a 6-point Likert scale, and detailed information on each item is provided in Supplementary Table S1. The content validity of the behavior status instrument was established by a group of experts, including a professional cardiologist and a nursing professor. The experts rated the instrument using a 4-point scale for the percentage of items rated 3 or 4 out of 4 for the content validity index (CVI). The CVI has been reported as 0.92.

The WHO-5 Well-Being Index, a one-dimensional quality-of-life instrument, was used^27^. It was developed by the World Health Organization and comprised five positive statements: “I have felt cheerful and in good spirits,” “I have felt calm and relaxed,” “I have felt active and vigorous,” “I woke up feeling fresh and rested,” and “My daily life has been filled with things that interest me.” Respondents were asked to rate their experience with these statements over the past 2 weeks on a 6-point Likert scale ranging from 0 (not present) to 5 (constantly present). The score was transformed from 0 (worst possible well-being) to 100 (best possible well-being). Scores below 50 indicate poor emotional well-being, and 28 or below indicate depression^28^.

### Intervention

PLI was developed by a nurse researcher based on patient-centered intervention, which concentrates on the patient’s unique circumstances and preferences^29^. PLI consisted of nutrition and exercise education for hypertensive patients and was conducted through a 30-min one-on-one in-person education based on a booklet developed by the researchers. First, education was provided on dietary management and eating habits that are helpful for hypertensive patients using a booklet. A hypertension management diet was planned to focus on the patient's preferred diet. For example, for patients who prefer salty flavors, soy sauce is recommended instead of salt when cooking, and flavoring vegetables and spices, such as vinegar, lemon, walnuts, peanuts, ginger, garlic, and green onions, have been suggested as alternatives to salty flavors. For those who enjoy eating carbohydrates, a plan was made to switch to buckwheat, gluten-free bread, or mixed-grain rice. A personalized diet plan was designed for patients who preferred fish to meat, teaching them how to make low-salt fish dishes.

Next, we educated the patients about exercise management, and together, we created an exercise plan based on their favorite activities. For patients who enjoyed walking, we planned to walk briskly or jump rope for 30 min thrice weekly. Additionally, we created exercise plans that included stretching and aerobic exercises for swimming, cycling, badminton, and golf. We encouraged the patients to record their daily exercise activities at the back of the booklet to motivate them to exercise independently.

**Figure 3:**
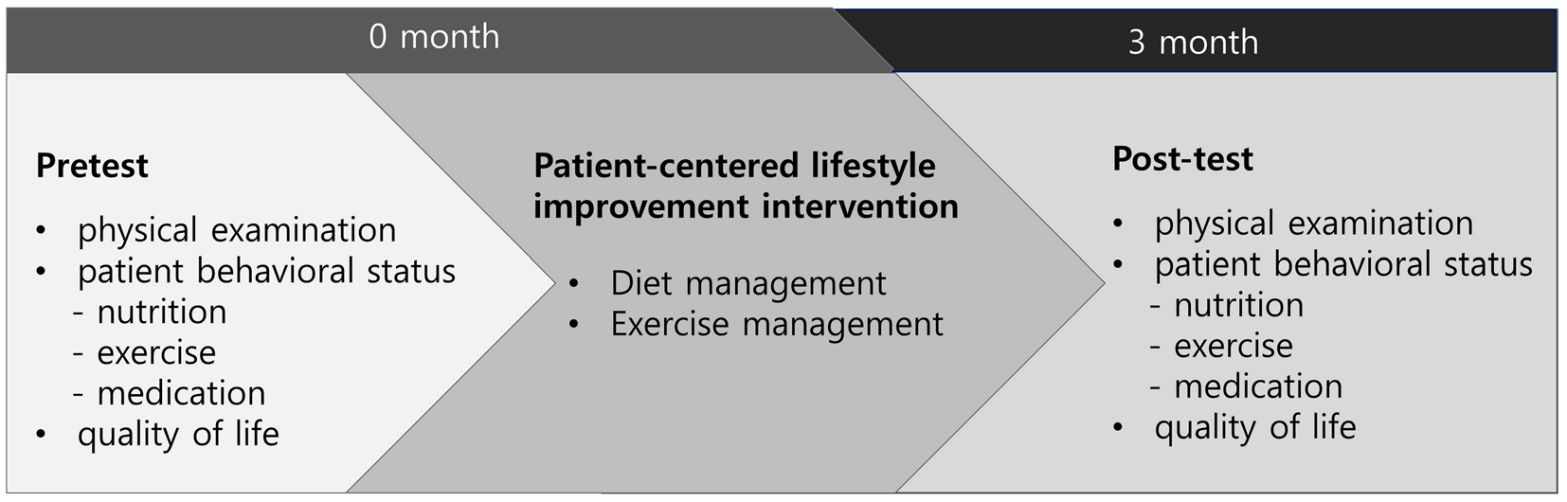
The study protocol.

Figure 3 shows the study protocol. Following a pretest survey on patient behavior and QoL, we provided patient-centered nutrition and exercise education tailored to each patient’s lifestyle. We performed blood tests, physical examination, and BP measurements as usual during outpatient follow-ups on the patients when they visited 3 months later for antihypertensive drug prescription. Posttest surveys were administered to evaluate changes in behavior and QoL.

### Study outcomes

BP responders to the patient-centered intervention were defined as those with differences of 10 mmHg or more than 10 mmHg for SBP or differences of 10 mmHg or > 10 mmHg for DBP. BP non-responders had differences of < 10 mmHg for SBP or < 10 mmHg for DBP.

### Statistical analyses

All categorical data are presented as frequencies and percentages, and continuous variables are expressed as means and standard deviations. For continuous variables, the Shapiro–Wilk test was used to confirm the normal distribution of each dataset. The Student’s *t*-test was used to compare normally distributed continuous variables, Pearson’s chi-squared test was used to compare categorical variables, and the Mann–Whitney U test was used to compare non-normally distributed continuous variables. The Wilcoxon signed-rank test was used to compare the variables measured at baseline and 3 months later. Furthermore, univariate and multivariate regression analyses were performed to assess the predictors of non-response to patient-centered interventions after adjustment for individual risk factors. Variables with predictive significance (*p* < 0.1) for non-response to patients centered intervention in the univariate analysis were included in the regression analysis. A *p*-value < 0.05 was considered statistically significant. All analyses were performed using the SPSS software (version 21.0; IBM Corp., Armonk, NY, USA).

### Supplementary Information

Supplementary Information.

## Data Availability

The datasets generated during the current study are available from the corresponding author on reasonable request.
