# Supplementary material for: Sex differences in effect of patients-centered intervention on blood pressure in patients with hypertension
Source: Sci Rep. 2023 Aug 25;13:13952. doi: 10.1038/s41598-023-41286-z (PMC10457352; doi:10.1038/s41598-023-41286-z)

Supplementary Materials

Sex Differences in Effect of Patients-Centered Intervention on Blood Pressure in Patients with Hypertension

Supplementary Table S1. Behavioral status questionnaire

Please read each sentence carefully then choose the option which best represents your opinion in the last 2 weeks.	All of the time<br>5	Most of the time<br>4	More than half of the time<br>3	Less than half of the time<br>2	Some of the time<br>1	At no time<br>0
1. I ate according to the plan in nutrition education for managing hypertension. (Ex) I ate brown rice, mixed grain rice, and rye bread instead of rice, noodles, bread, and rice cake.						
2. I used a recipe from nutrition education for managing hypertension.<br>(Ex) I ate boiled or poached food rather than oil-based food.						
3. I practiced the number of exercises as planned in exercise education for hypertension management.<br>(Ex) walking for an hour 3 times a week						
4. I practiced the exercise method planned in exercise education for hypertension management.<br>(Ex) walking, swimming, cycling, tennis, etc.)						
5. I took my high blood pressure medicine at the same time every day.						
6. I took the correct dose of my high blood pressure medicine.						

Supplementary Table S2. Gender difference in behavioral and quality of life changes

	All (n =95)	Men (n =55)	Women (n =40)	P -value
Behavior status				
Diet, baseline	4.18±2.71	3.85±2.72	4.63±2.68	0.173
Diet, 3month	8.25±1.79	7.87±1.99	8.78±1.31	0.014
Difference in Diet	4.07±3.00	4.02±2.90	4.15±3.18	0.834
Exercise, baseline	3.27±3.17	3.38±3.26	3.13±3.07	0.699
Exercise, 3month	7.60±2.51	7.36±2.63	7.93±2.33	0.284
Difference in exercise	4.33±3.30	3.98±3.36	4.80±3.19	0.470
Medication, baseline	7.83±2.20	7.35±2.65	8.50±1.09	0.005
Medication, 3month	9.78±0.80	9.75±0.87	9.83±0.71	0.635
Difference in medication	1.95±2.38	2.40±2.79	1.33±1.46	0.069
Behavior total*, baseline	15.28±5.52	14.58±5.75	16.25±5.11	0.147
Behavior total*, 3 month	25.63±3.90	24.98±4.16	26.53±3.35	0.056
Difference in Behavior total*	10.35±6.20	10.40±6.23	10.28±6.23	0.923
Quality of life				
QOL, baseline	9.83±6.13	9.98±6.23	9.63±6.07	0.781
QOL, 3month	19.93±3.66	20.33±3.59	19.38±3.73	0.213
Difference in QOL	10.09±6.59	10.35±6.47	9.75±6.82	0.666

QOL, quality of life

Supplementary Figure S1. Changes in body mass index and lipid profile after the patients-centered intervention

(A) Changes in body mass index after 3month in men and women.

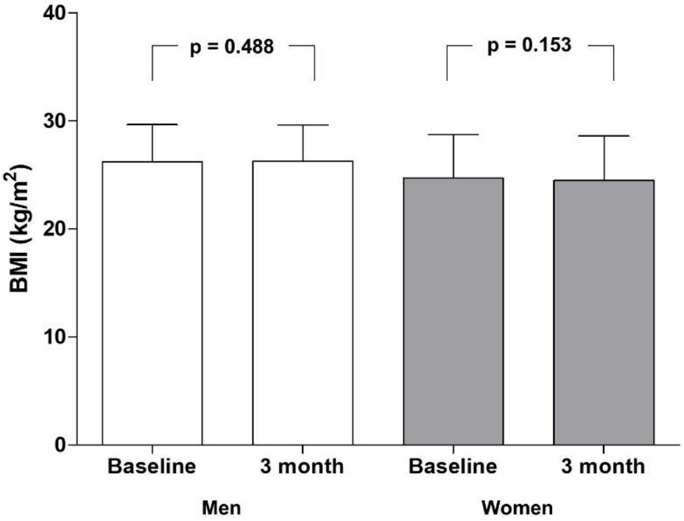

(B) Changes in total cholesterol after 3month in men and women

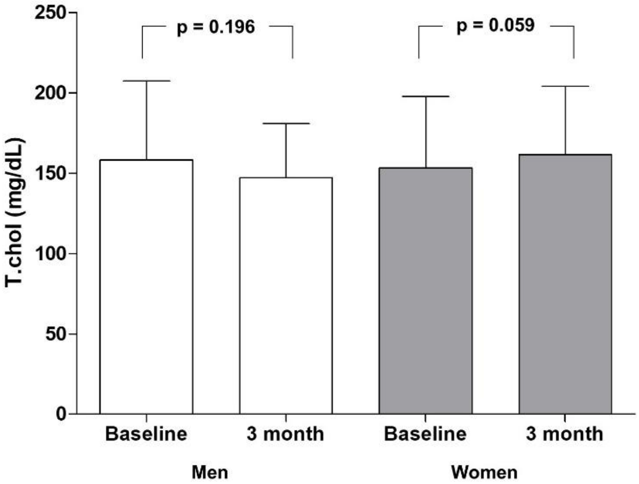

(C) Changes in low-density lipoprotein cholesterol after 3month in men and women

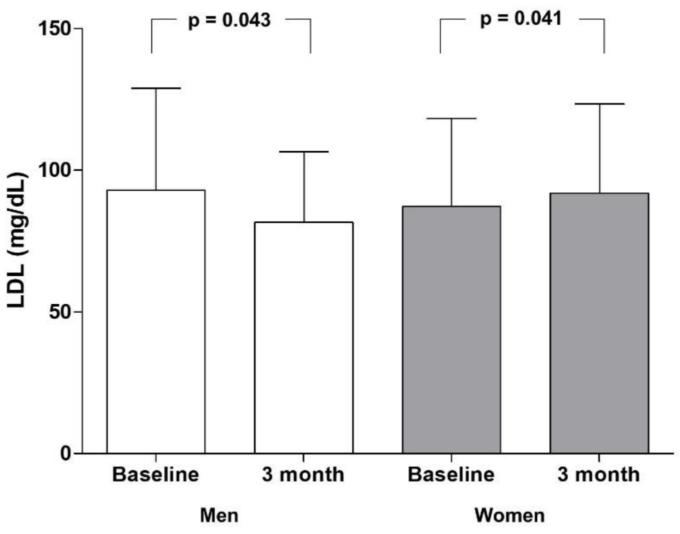

(D) Changes in triglyceride after 3month in men and women

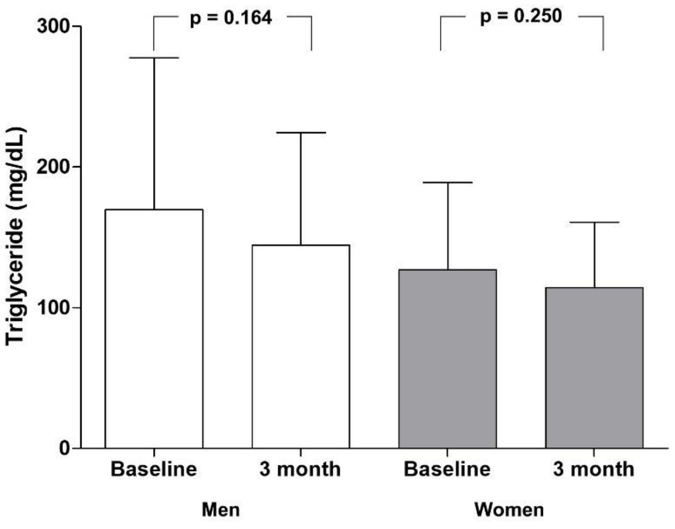

(E) Changes in high-density lipoprotein cholesterol after 3month in men and women

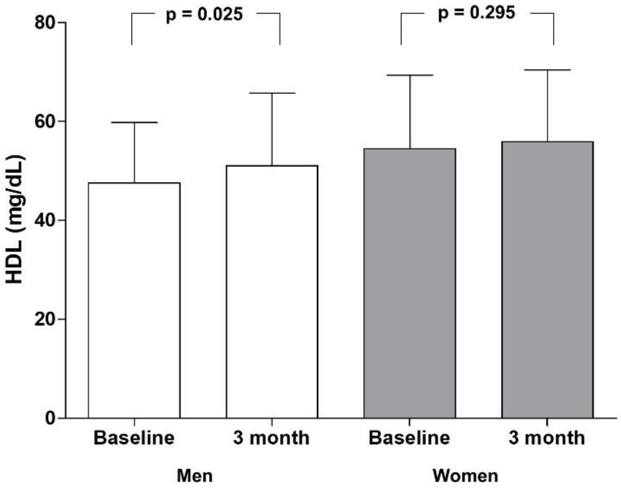

Supplement: Supplementary file 1 — Supplementary Information. [file 41598_2023_41286_MOESM1_ESM.pdf]
